# Selection of the Optimal Smart Meter to Act as a Data Concentrator with the Use of Graph Theory

**DOI:** 10.3390/e23060658

**Published:** 2021-05-24

**Authors:** Piotr Kiedrowski

**Affiliations:** Institute of Telecommunications and Computer Science, UTP University of Science and Technology in Bydgoszcz, Al. Prof. S. Kaliskiego 7, 85-796 Bydgoszcz, Poland; pkied@utp.edu.pl

**Keywords:** graph theory, smart meter, smart metering, wireless sensor network

## Abstract

Changing the construction of mart Meter (SM) devices, more specifically equipping them with more than one communication module, facilitates the elimination of a Transformer Station Data Concentrator (TSC) module, moving its function to one of the SMs. The opportunity to equip a chosen device in an additional communication module makes it possible to use it as an acquisition node. The introduction of this solution creates a problem with the optimum selection of the above-mentioned node out of all the nodes of the analyzed network. This paper suggests the criterion of its location and, as per the criterion, the way of conduct using the elements of the graph theory. The discussion is illustrated with the examples of the possibility to use the method for the optimization of the architecture of the network. The described method makes it possible to choose the location of a backup acquisition node as well as locate intermediary nodes (signal repeaters) in case of a failure (removal) of some SM devices. In the era of the common introduction of dispersed telemetric systems requiring an adequate level of performance and reliability of information transmission, the offered method can be used for the optimization of the structures of Smart Grids.

## 1. Introduction

Currently, a rapid growth of technologies based on computers with increasingly good parameters, a progressing miniaturization of electronic systems and the development of professional software and methods of information transfer are being observed. Wireless transmission technologies are becoming more and more important thanks to their low cost and quick deployment. Amongst them are wireless sensor networks (WSNs) [1]. They feature low transmitting power, very low energy consumption, are operated with the most recent monolithic transceivers, and their nodes cooperate directly with sensors of various measurable parameters.

The networks are widely used, for example, in municipal buildings as early warning systems for terrorist attacks or environmental contamination [2], for monitoring the environment (temperature, humidity, pollution level) [3], for industrial surveillance (providing information about the overall condition of machines used in the production process, e.g., vibration, fluid level, temperature of components, etc., received from sensors placed in hard-to-reach or inaccessible places) [4], for the control of traffic [5], and in medicine (allowing higher effectiveness of diagnosing and monitoring patients’ health without the need to connect them to medical devices with limited mobility, e.g.,wireless body sensor networks (WBSNs)) [6,7]. WSNs areused for the creation of smart cities, professionally known as Cyberville, Digital City, or Electronic Communities. Their most important features are digital and computerized management of energy production and distribution;, automatic smart security systems; lighting and heating control; management of car parks; fast data transmission using the latest wireless technologies in the 5G network; municipal serviceless meters and usage sensors with digital water, electricity, and gas distribution management systems; smart medical care systems remote life quality management and improvement such as systems of e-participation in various forms of cultural, sporting, leisure, and social activity; and surveillance systems, biometric systems, and safety systems for terrorist and criminal threats, etc. [8,9,10].

An example of the utilization of the WSN technology is the wireless scattered telemetry network intended for the last-mile service of the Smart Metering system. The term Smart Metering refers to smart power grids which provide the communication between producers and recipients of energy as well as with power repositories. The basic component of these networks is an expanded metering systemthat gives access to information about the power consumption. It consists of ICT systems transferring the measurements to decision points, as well as smart information, forecasting, and decision algorithms. The Smart Metering system makes it possible to transfer and process information important for a power grid, such as the power consumption by receivers and the production of energy from conventional and renewable sources. It ensures a high flexibility level of the power grid which in turn allows it to control the demand and supply of power quickly and optimally [11].

A data concentrator collecting the information from Smart Meter (SM) devices is one of the basic components of ICT systems [9]. Usually, it is installed next to an MV/LV transformer, hence its name Transformer Station Data Concentrator (TSC) [12]. TSC has the same role as an acquisition node in sensor networks. Similarly to sensor networks, in order to increase the range of the last-mile network operation, a multi-hop technique is used [13].

In the communication between the two above-mentioned components of the system, two basic transmission methods are used, wired and wireless. Amongst the wireless technologies, the most frequently used are radio transmission in the Industrial-Scientific-Medical (ISM) band (known as RF) and General Packet Radio Service (GPRS), available as one of the services offered by the Global System for Mobile Communications (GSM). SM devices equipped with GPRS communication modules are used only in countries with low population density such as Denmark or Sweden [13,14]. If the density is higher, the GPRS modules are installed only in TSCs, thus giving them access to IP networks.

Amongst the wired technologies, another two main techniques can be mentioned. These are Power Line Communication (PLC) and M-bus. The M-bus technology, designed for the reading of various kinds of metering devices, is used in Smart Metering only in last-mile network with direct hubs—Local Metering Concentrators (LMCs)—to which multiple SM devices can be connected through the use of M-bus [9,15,16]. LMC communicates with TSC by the use of the RF or the PLC technology. Modern SMs are equipped with M-buses and/or Modbus interfaces, which facilitate the installation of a few communication modules in one meter [17] thanks to the ongoing miniaturization of components, and equips the SMs in more than one LMC unit whereby each of them can use a different communication technique. Adopting such solutions makes it possible to realize and utilize last-mile networks more flexibly while increasing their security, reliability, quality, productivity, and the ease of migration process, e.g., from RF to PLC [18,19,20,21].

This article touches upon a new approach in the last-mile network structural solutions, i.e.,the elimination of TSC and moving its functions to an SM equipped with two LMC units, namely RF or PLC and GSM. The benefits of introducing this solution are lower network creation costs, the elimination of additional TC–SM connections, which are often unreliable due to the long distance between a transformer and an edge of a last-mile network, and there is no need for the maintenance of TSCs involving complex procedures, assuring the safety of transmission and service. However, moving functions from TSC to SM, apart from the above-mentioned benefits, also causes a problem—a solution for which the author offers in this paper. The solution consists of choosing a parameter of the optimal SM localization, which additionally works as a data concentrator.

## 2. Research Method

For the analysis of telemetric networks, parts of the graph theory were used [22]. The topology of such networks can be defined by a formula describing geometrical random graphs [9]:

(1) G=(V,E,R)

where *V* is a set of vertices, *E* is a set of edges, and *R* is set of radii determining the transmission range.

**Figure 1:**
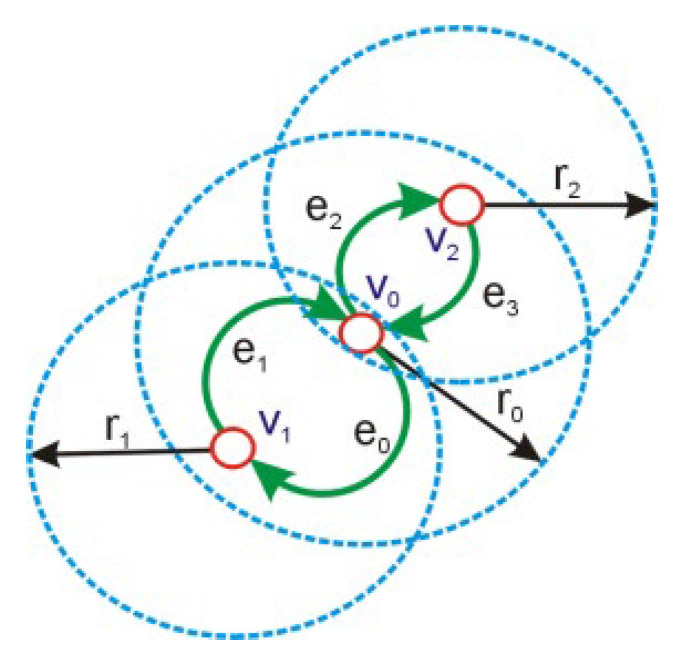
An example of a graph describing the topology of a network.

Each *v_i_* vertex of the *G* graph represents a single network node. Each node is surrounded by a circle of radius *r_i_* depicting the range of the signal emitted by a node’s transmitter. All nodes in the circle surrounding the node (assuming omnidirectional antennas are used) can freely communicate with the surrounded node bidirectionally and are considered directly adjacent, as it is shown in Figure 1.

The below formula [23] defines a set of nodes in the transmission range of a given node:

(2) $$Br(x)=\left\{y:\left|x-y\right|<r\right\},\;x,y\in V$$

where *Br*(*x*) is a set of nodes within the transmission range, *x* and *y* are the localization of the nodes, and *r_i_* is the radius of the signal range.

The edge set *E* contains all edges of the *G* graph and represents direct paths connecting any chosen vertices and all their adjacent items within the circle, representing the communication range of each node.

It is assumed that the edges do not have to represent bidirectional connections, which means that the presence of edges (*x*,*y*) does not mean that edges (*y*,*x*) exist. The assigned measurable value between two vertices of a graph, such as distance, angle, and amount of energy in the node or probability, can be assigned to the edge as its weight, provided that it can have different values for edges (*x*,*y*) and (*y*,*x*). The definition of asymmetric edges is expressed with the Formula (3):

(3) $$E=\left\{\left(x,y,d\right):y\in Br(x)\wedge d=\left|x-y\right|\right\},\;x,y\in V$$

where *E* is a set of asymmetric edges and *d* is the measure of the asymmetry of weights.

A graph obtained this way, describing the whole connection topology of the network, is called a maximum power graph (in real-life conditions when first determining the connection topology, the transmitters work with maximum power). The structure of this graph has to be reduced because of the presence of many redundant connections, which are undesirable due to their transmitting of identical information, leading to collisions, unnecessary power consumption, and the increase in the final network emissivity. The reduction in these connections leads to the creation of a minimum spanning tree whose root is an acquisition node in which all the information consistent with the network’s function are collected.

**Figure 2:**
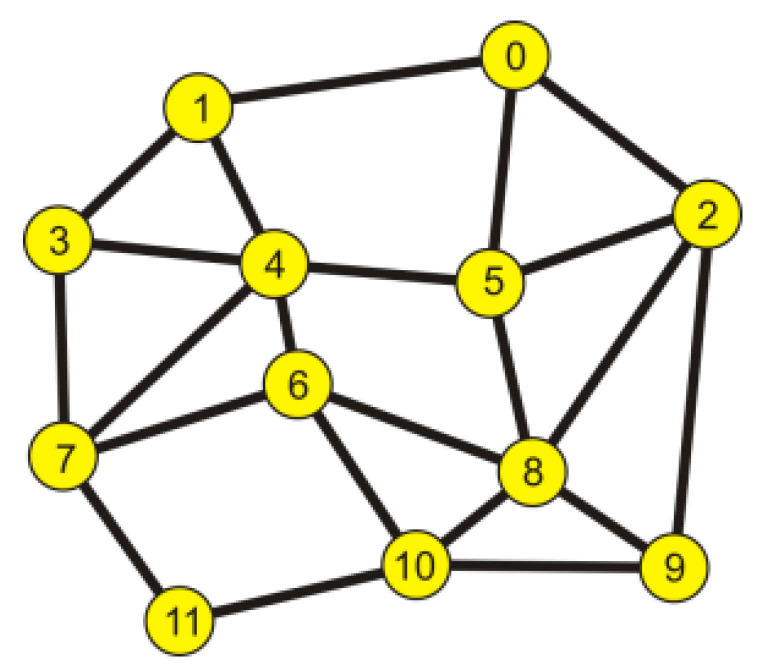
An example of a telemetry network.

The configuration of the discussed network can be presented with a graph with an adjacency matrix [24]. To explain the way of conduct, a graph describing an example of a virtual network is shown in Figure 2. The edges of the graph correspond to the links connecting its nodes—that is, the nodes intermediary to the transmission and acting commutatively.

The network shown in Figure 2 is described by an adjacency matrix (*Ms*):

$$\left[Ms\right]=\left[\begin{array}{cccccccccccc} 0 & 1 & 1 & 0 & 0 & 1 & 0 & 0 & 0 & 0 & 0 & 0\\ 1 & 0 & 0 & 1 & 1 & 0 & 0 & 0 & 0 & 0 & 0 & 0\\ 1 & 0 & 0 & 0 & 0 & 1 & 0 & 0 & 1 & 1 & 0 & 0\\ 0 & 1 & 0 & 0 & 1 & 0 & 0 & 1 & 0 & 0 & 0 & 0\\ 0 & 1 & 0 & 1 & 0 & 1 & 1 & 1 & 0 & 0 & 0 & 0\\ 1 & 0 & 1 & 0 & 1 & 0 & 0 & 0 & 1 & 0 & 0 & 0\\ 0 & 0 & 0 & 0 & 1 & 0 & 0 & 1 & 1 & 0 & 1 & 0\\ 0 & 0 & 0 & 1 & 1 & 0 & 1 & 0 & 0 & 0 & 0 & 1\\ 0 & 0 & 1 & 0 & 0 & 1 & 1 & 0 & 0 & 1 & 1 & 0\\ 0 & 0 & 1 & 0 & 0 & 0 & 0 & 0 & 1 & 0 & 1 & 0\\ 0 & 0 & 0 & 0 & 0 & 0 & 1 & 0 & 1 & 1 & 0 & 1\\ 0 & 0 & 0 & 0 & 0 & 0 & 0 & 1 & 0 & 0 & 1 & 0 \end{array}\right]$$

By exponentiating the (*Ms*) matrix, we obtain a set of paths created by the edges connecting the graph nodes, whose length corresponds to the power of the matrix. The process is carried on until it is confirmed that all the nodes are interconnected and the maximum power of the (*Ms*) matrix is the diameter of the analyzed graph. By the analysis of the obtained components of the matrix, minimum lengths of the paths connecting chosen nodes (created by the smallest number of edges) are determined, and their number is calculated, as well.

Table 1 includes the calculated lengths of minimum paths connecting the nodes of the analyzed graph, and Table 2 shows the numbers of these paths.

For example, there are four minimum paths connecting node 4 with node 9, which consist of three edges.

**Figure 3:**
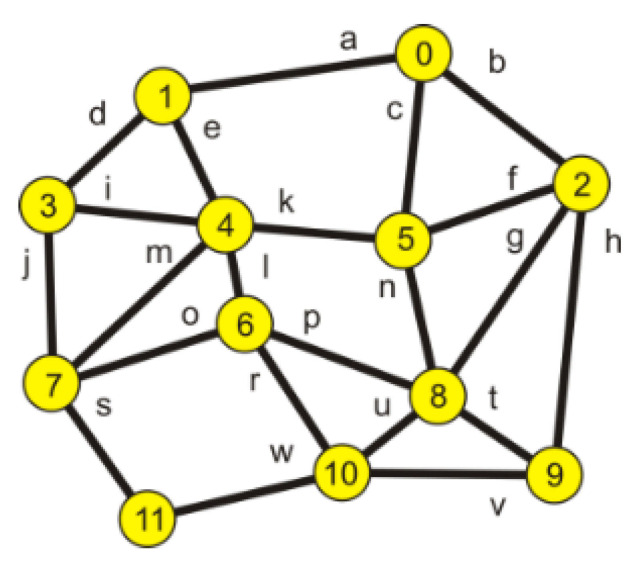
Edge marking.

However, on the basis of the obtained results, it is not possible to determine which edges of the set create individual paths. To define a configuration of every path, each edge is given a name, presented in Table 3 and in Figure 3.

By performing the exponentiation of the sign matrix according to the rule of matrix exponentiation, sets of minimum paths are obtained together with their structure. It is shown in Table 4.

While reviewing the components in Table 4, it is clearly visible that the numbers and the lengths of the minimum paths connecting the nodes are consistent with the calculation results presented in Table 1 and Table 2.

## 3. Determining the Optimal Position for the Acquisition Node of a Telemetry Network

So far, the analysis was made with the assumption that the probability of realization of a correct transmission by each node is 1, which is in contradiction to reality, and the parameter is especially important for radio networks.

It is known that radio links are less resistant to external interference than fiber-optic or cable links, and a radio wave carrying information is subject to suppression depending on the distance between a transmitter and a receiver. The dependency is partially described by the free space signal suppression value formula *FSL* (Free Space Loss):

(4) $$FSL=32.44\;\mathrm{dBm}+20\log\left(f\right)+20\log\left(d\right)$$

where *f* is the transmission frequency in MHz and *d* is the distance between a transmitter and a receiver in km [25].

**Figure 4:**
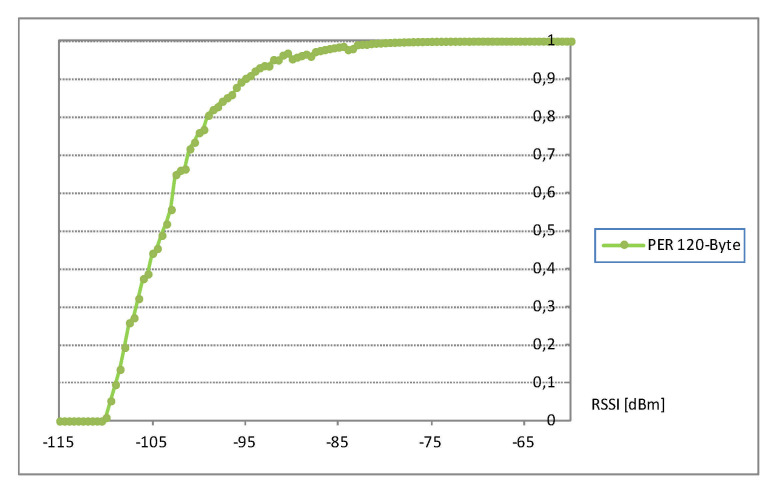
The chart of the dependency of link quality in the function of the level of the signal reaching the receiver.

By assuming the same level of radio signal emission, e.g., 0 dBm, the same sensitivity of the receivers and the use of omnidirectional antennas (which causes the increase in the suppression value by 40 dBm of the signal reaching the receiver), the parameter RSSI (Received Signal Strength Indicator) was determined on the basis of the dependency shown in Figure 4, taken from paper [26], and PER (Packet Error Ratio) parameter values were found (it was assumed that 433 MHz radio frequency was used).

In the analyzed case, assuming the distances between the nodes are as shown in Table 5 (they are correlated with the data presented in Figure 1), the RSSI and PER values were determined.

Table 5 includes the RSSI values resulting from the distance between the network nodes linearly. The possibility that suppressing signals for a given link can vary for individual directions of information transmission was not taken into consideration.

To check the construction of the structure after including different signal suppression depending on the information transfer direction, it was assumed that the values of the probability of a return transmission from the target nodes to the source nodes reaches the values given in Table 6 (the ‘←’ sign means the change of the information transfer direction).

**Figure 5:**
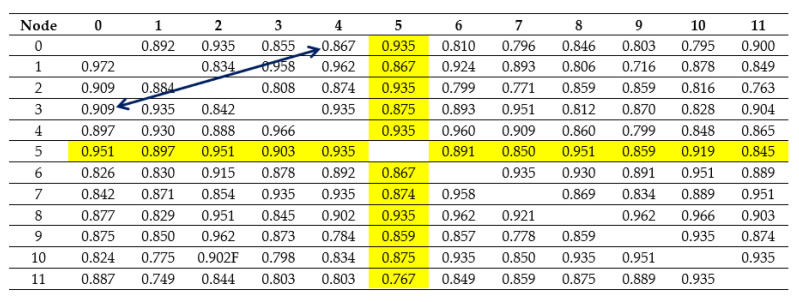
The calculated probability values of obtaining a correct transmission.

By using the data from Table 6 and assuming that the data of the information transfer direction from the source nodes to the target nodes are above the diagonal of the table, and from the target nodes to the source nodes are below the diagonal, the probability values were calculated and are presented in Figure 5.

The way of conduct described in the previous part of the paper was used for the determination of the optimal position of the acquisition node of the network, i.e., the node whose average probability of the realization of a correct transmission is the highest in comparison with the other nodes. For this purpose, the probability values for both transmission values were multiplied. They corresponded to individual paths connecting the nodes of the graph (for example, nodes 0 and 5) and the obtained values were averaged. It is shown in Table 7.

On the basis of the data presented in the table above, it was concluded that the optimal localization for an acquisition node was node 4.

Table 8 includes the set of minimum length paths, taking into consideration the presence of parallel, internodal paths (the parallel paths are the paths consisting of the same number of edges connecting the same nodes—source and target).

**Figure 6:**
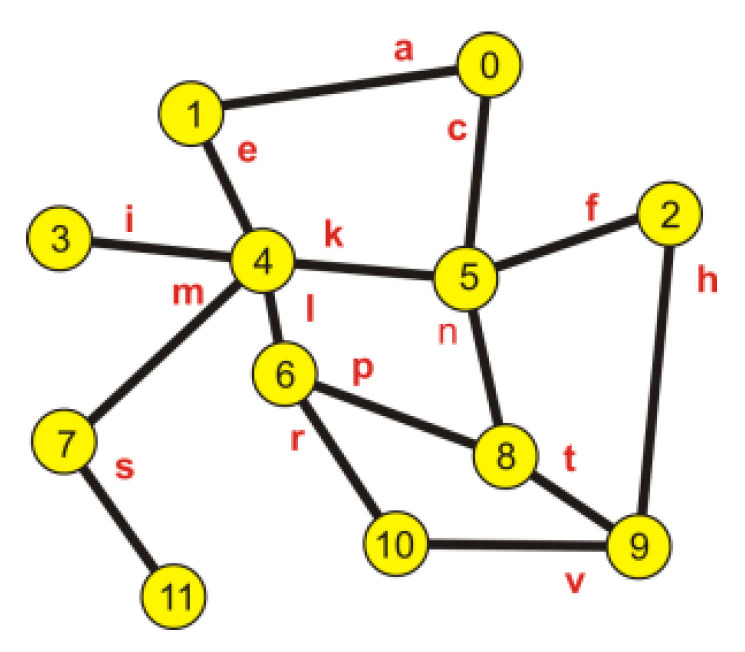
The image of the obtained graph.

In Figure 6, the image of the obtained graph describing the network created by the minimum length paths is shown.

Thanks to the analysis of the elements in Table 8, a minimum spanning tree was created; its root is node 4, so by removing the redundant connections, paths were chosen that ensured the highest probability of a correct transmission. The set of paths creating the minimum spanning tree is included in Table 9.

**Figure 7:**
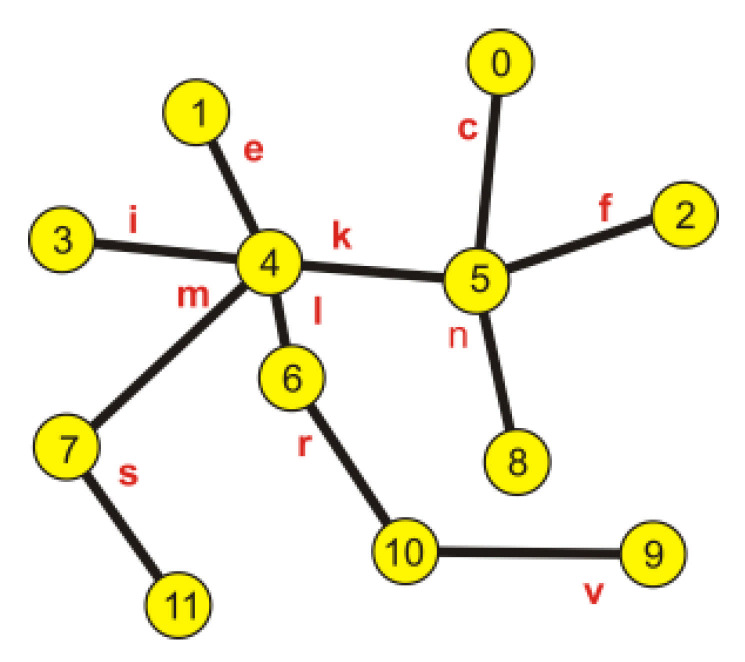
The image of a minimum spanning tree.

In Figure 7,the obtained minimum spanning tree is shown.

The described way of conduct can be used for the analysis of a situation caused by a failure of individual links due to which the graph describing the obtained tree becomes a disconnected graph resulting in isolated nodes. When there are two parallel paths connecting chosen nodes created by various combinations of edges (in the examples 4—0, 4—8, and 4—9), the graph stays connected. Thanks to the proposed method, that is, the analysis of the matrix powers (*Ms*), it is possible to determine a set of emergency connections and avoid a connection shortage. The set of paths used in case of a link failure is included in Table 10.

The determined set of paths can be saved in routing memories of individual nodes and used to support transmission in case of a failure of certain links.

## 4. The Analysis of the Possibility to Ensure the Operation of a Network after Node Removal

In practice, due to changes in networks (for example, by replacing the energy meters with devices working in a different technology), ‘holes’ appear, which causes the graphs describing these networks to be disconnected. In such cases, a signal repeater is often installed in place of one of the nodes. Its task is only to mediate in the transmission of signals between sensors and an acquisition node. Using such a solution is led by the use of an already existing infrastructure enabling the supply of power to the repeater. There is also a case of choosing a location for the repeater. In this case, the proposed method of network analysis can work as well.

**Figure 8:**
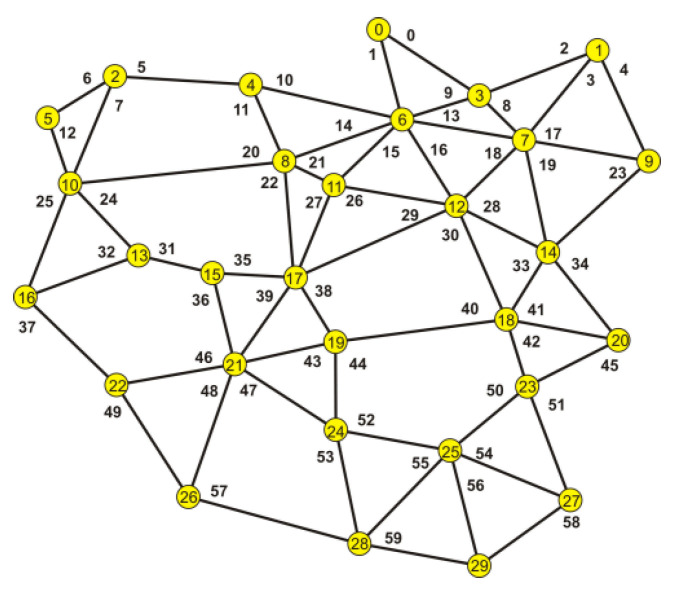
The analyzed network before nodes failure.

To explain the accepted way of conduct, the analysis of a more complex network was used. The network is shown in Figure 8.

By following the rules described above, the optimum location of the acquisition node was determined to be node 17.

**Figure 9:**
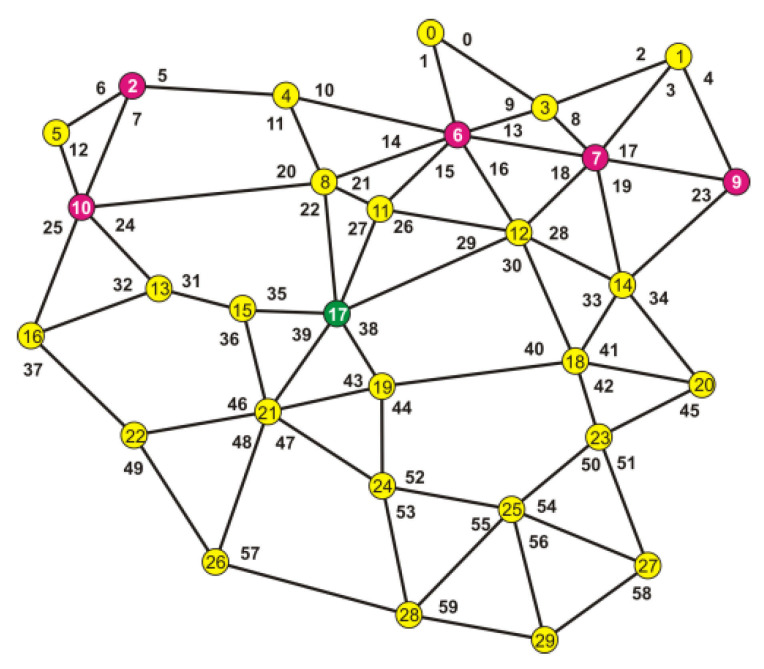
An image of the network after the nodes failure.

It was assumed that for some reasons, nodes 2, 6, 7, and 10 had been excluded which caused nodes 0,1, 3, and 5 to lose the possibility to contact the acquisition node. This is shown in Figure 9.

**Figure 10:**
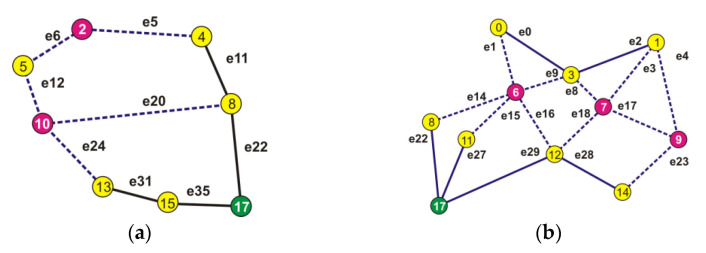
The structures after the exclusion of the chosen nodes. (**a**) related to the fifth node; (**b**) associated with the remaining nodes i.e., 0, 1 and 3.

By analyzing the changed structure of the network, it was divided into two parts. One related to the fifth node (Figure 10a) and one associated with the remaining nodes 0, 1, and 3 (Figure 10b).

The sets of virtual shortest paths connecting the isolated nodes are given in Table 11.

The data in Table 11 show that when it comes to node 5, it is clear for a signal repeater to be installed in node 10.

The situation presented in Figure 10b is more difficult to analyze. In that case, it should be verified which of the edges creating the paths ensure obtaining the maximum probability of a correct transmission.

Even a superficial scan of the network scheme shows that placing a repeater in node 9 is futile, so the focus was moved towards node 6 or 7. The set of minimum length paths for nodes 0, 1, and 3 is presented in Table 12.

The configurations of paths connecting individual nodes with the acquisition node ensuring the highest probability of a successful transmission were analyzed: from node 0—e1, e15, e27; from node 1—e2, e9, e15, e27; from node 3—e9, e15, e27. Each of the above-mentioned paths is connected with node 6, so it is therefore the location of an intermediary node.

## 5. Discussion

The proposed method allows for the determination of the best location ofan SM device for an additional role as a concentrator, thanks to which it is possible to eliminate a TSC module as well as redundant connections between the TSC and its adjacent SM modules. The absence of need for the installation of TSC lowers the cost of the network construction and its location maintenance (that is a transformer station MV/LV). It leads to the avoidance of time-consuming, complex, and expensive procedures. The removal of unreliable links between the TSC and the SM modules, resulting from a long distance between them, causes the improvement of the reliability and traffic parameters of the network. Improvement of the traffic parameters allows for more frequent reading, which is crucial in energy consumption forecasting. One additional benefit is the possibility to perform more frequent reading of the meters, which is particularly crucial, for example, in Smart Grid systems, with distributed generation, increase in the number of SMs in a last-mile network, and therefore the extension of the operating area of the network.

Sometimes, in order to improve the traffic parameters of a network, it is necessary to divide it into several subnetworks. The division of networks within the structure using TSC was possible only if the main network was extended with a new transformer. The proposed method makes it possible to determine the best location for the SMs, which are also supposed to work as concentrators in particular subnetworks. The paper also proves that the proposed method can be used for finding a place for backup concentrators and repeaters.

## 6. Conclusions

All the presented solutions referred to last-mile networks using the RF technology. Thanks to the introduction of the ITU-T recommendations defining the PLC interfaces for Smart Metering [27,28,29], the technology can be used in many areas of a Smart Grid [30]. Currently, the author is working on the adaptation of the proposed method for networks based on the G3 PLC [28] and PRIME [29] interfaces. In this case, it is necessary to develop a method for the calculation of the PER values for the links using PLC (not only RSSI, but also Signal to Noise Ratio (SNR) and a way to describe the topology of a network (in case of a three-phase mains, the installed SM devices for transmission can use three common media or only one of them).

## Figures and Tables

**Table 1:** The lengths of the minimum paths (the number of edges).

1	1										
2	1	2									
3	2	1	3								
4	2	1	2	1							
5	1	2	1	2	1						
6	3	2	2	2	1	2					
7	3	2	3	1	1	2	1				
8	2	3	1	3	2	1	1	2			
9	2	3	1	4	3	2	2	3	1		
10	3	3	2	3	2	2	1	2	1	1	
11	4	3	3	2	2	3	2	1	2	2	1
Node	0	1	2	3	4	5	6	7	8	9	10

**Table 2:** The numbers of the minimum paths.

1	1										
2	1	1									
3	1	1	2								
4	2	1	1	1							
5	1	2	1	1	1						
6	4	1	1	2	1	2					
7	3	2	2	1	1	1	1				
8	2	4	1	3	2	1	1	1			
9	1	1	1	8	4	2	2	3	1		
10	3	1	2	3	1	1	1	2	1	1	
11	6	2	2	1	1	2	2	1	1	1	1
Node	0	1	2	3	4	5	6	7	8	9	10

**Table 3:** Adopted edge marking.

Edge	a	b	c	d	e	f	g	h	i	j	k
Nodes	0–1	0–2	0–5	1–3	1–4	2–5	2–8	2–9	3–4	3–7	4–5
Edge	l	m	n	o	p	r	s	t	u	v	w
Nodes	4–6	4–7	5–8	6–7	6–8	6–10	7–11	8–9	8–10	9–10	10–11

**Table 4:** Minimum paths configurations.

Node	0	1	2	3	4	5	6	7	8	9	10	11
0	-	a	b	ad	ae ck	c	ael ckl cnp bgp	adj ckm aem	cn bg	bh	bhv cnu bgu	adjs aems cnuw bguw bhvw ckms
1	a	-	ab	d	e	ek ac	el	dj em	elp ekn acn abg	abh	elr	djs ems
2	b	ab	-	bad fki	fk	f	gp	fkm gpo	g	h	hv gu	hvw guw
3	ad	d	bad fki	-	i	ik	il jo	j	ilp jop ikn	ilpt iknt ikfh ikrv jorv jopt jswv dabh	ilr jor jsw	js
4	ae ck	e	fk	i	-	k	l	m	Lp kn	lpt lrv knt kfh	lr	ms
5	c	ek ac	f	ik	k	-	kl np	km	n	nt fh	nu	nuw kms
6	ael ckl cnp bgp	el	gp	il jo	l	kl np	-	o	p	pt rv	r	rw os
7	adj ckm aem	dj em	fkm gpo	j	m	km	o	-	op	opt swv	or sw	s
8	cn bg	elp ekn acn abg	g	ilp jop ikn	lp kn	n	p	op	-	t	u	uw
9	bh	abh	h	ilpt iknt ikfh ikrv jorv jopt jswv dabh	lpt lrv knt kfh	nt fh	pt rv	opt swv	t	-	v	vw
10	bhv cnu bgu	elr	hv gu	ilr jor jsw	lr	nu	r	or sw	u	v	-	w
11	adjs aems cnuw bguw bhvw ckms	djs ems	hvw guw	Js	ms	nuw kms	rw os	s	uw	vw	w	-

**Table 5:** The determined RSSI and PER values.

*Edge*	a	b	c	d	e	f	g	h	i	j	k
*d(m)*	48.6	36.45	34.02	29.16	26.73	36.45	51.03	55.89	34.02	33.04	34.02
*RSSI* *(dBm)*	−95.38	−92.88	−92.28	−90.94	−90.19	−92.88	−95.80	−96.59	−92.28	−92.03	−92.28
*PER*	0.8922	0.9347	0.9354	0.9579	0.9620	0.9347	0.8592	0.8592	0.9354	0.9505	0.9354
*Edge*	l	m	n	o	p	r	s	t	u	v	w
*d(m)*	19.44	43.74	31.59	37.91	38.88	33.05	31.59	26.73	23.33	38.88	36.45
*RSSI* *(dBm)*	−87.42	−94.47	−91.64	−93.22	−93.44	−92.03	−91.64	−90.19	−89.01	−93.44	−92.88
*PER*	0.9723	0.9092	0.9513	0.9347	0.9299	0.9505	0.9513	0.9620	0.9657	0.9347	0.9347

**Table 6:** The assumed RSSI and PER values for the second transmission direction.

Edge	*← a*	*← b*	*← c*	*← d*	*← e*	*← f*	*← g*	*← h*	*← i*	*← j*	*← k*
PER	0.9723	0.9092	0.9513	0.9347	0.9299	0.9505	0.9513	0.9620	0.9657	0.9347	0.9347
Edge	*← l*	*← m*	*← n*	*← o*	*← p*	*← r*	*← s*	*← t*	*← u*	*← v*	*← w*
PER	0.8922	0.9347	0.9354	0.9579	0.9620	0.9347	0.8592	0.8592	0.9354	0.9505	0.9354

**Table 7:** The resulting probability of obtaining a correct transmission.

Node	0	1	2	3	4	5	6	7	8	9	10	11	*P_awr_*
0		0.867	0.850	0.777	0.777	0.890	0.669	0.670	0.743	0.702	0.656	0.562	0.7421
1	0.867		0.737	0.895	0.895	0.777	0.776	0.778	0.670	0.783	0.689	0.636	0.7931
2	0.850	0.737		0.681	0.777	0.888	0.731	0.657	0.817	0.827	0.736	0.644	0.7587
3	0.777	0.895	0.681		0.903	0.790	0.790	0.888	0.705	0.586	0.679	0.726	0.7655
4	0.777	0.895	0.777	0.903		0.874	0.868	0.850	0.777	0.653	0.771	0.695	0.8035
5	0.890	0.777	0.888	0.790	0.874		0.777	0.743	0.890	0.735	0.804	0.655	0.8021
6	0.669	0.776	0.731	0.790	0.868	0.777		0.895	0.895	0.764	0.888	0.754	0.8001
7	0.670	0.778	0.657	0.888	0.850	0.743	0.895		0.801	0.648	0.755	0.817	0.7731
8	0.743	0.670	0.817	0.705	0.777	0.890	0.895	0.801		0.827	0.903	0.790	0.8016
9	0.702	0.783	0.827	0.586	0.653	0.735	0.764	0.648	0.827		0.888	0.777	0.7446
10	0.656	0.689	0.736	0.679	0.771	0.804	0.888	0.755	0.903	0.888		0.874	0.7859
11	0.562	0.636	0.644	0.726	0.695	0.655	0.754	0.817	0.790	0.777	0.874		0.7209

**Table 8:** The set of minimum length paths obtained with the assumption that the acquisition node is node 4.

Node	0	1	2	3	5	6	7	8	9	10	11
Set of Paths	ae ck	E	fk	I	K	l	m	lp kn	lpt lrv knt kfh	lr	*ms*
*P_path_*	0.777	0.895	0.777	0.903	0.874	0.868	0.850	0.777	0.653	0.771	0.695

Where: Set of Paths, the set of all minimum length paths; *P_path_*, the probability of the realization of transmission between nodes.

**Table 9:** The set of paths creating the minimum spanning tree.

Node	0	1	2	3	5	6	7	8	9	10	11
*C_min_*	ck	E	fk	i	K	l	M	nk	vrl	rl	sm
*P_pathtree_*	0.778	0.895	0.777	0.903	0.874	0.868	0.850	0.778	0.685	0.771	0.695

Where: *C_min_*, the configuration of a minimum path; *P_pathtree_*, the probability of the realization of transmission.

**Table 10:** The set of paths used in case of a link failure.

Link Failure	Node	0	1	2	3	5	6	7	8	9	10	11
E	Sets of Paths	ck	Di	fk	I	K	l	m	nk	vrl	rl	sm
F		ck	E	bck	I	K	l	m	nk	vrl	rl	sm
K		ae	E	bae	I	Cae	l	m	lp	vrl	rl	sm
I		ck	E	fk	Jm	K	l	m	nk	vrl	rl	sm
L		ck	E	fk	I	K	om	m	nk	knt	unk	Sm
M		ck	E	fk	I	K	l	ol	nk	vrl	unk	Wrl
R		ck	E	fk	I	K	l	m	nk	knt	unk	Sm
S		ck	E	fk	I	K	l	m	nk	vrl	rl	Wrl

**Table 11:** The sets of the shortest paths (for the node 5).

Node	Paths	*P_path_*
5	e12, e20, e11	0.6570
	e6, e5, e11, e22	0.6706
	e12, e24, e31, e35	0.7900

**Table 12:** The set of minimum length paths (nodes 0, 1, 3).

Node	Paths	*P_path_*
0	e1, e15, e27	0.78192
	e1, e16, e29	0.68325
	e1, e14, e22	0.72990
1	e3, e18, e29	0.66908
	e2, e9, e15, e27	0.71395
	e2, e9, e14, e22	0.66645
3	e9, e15,e27	0.81298
	e9, e14,e22	0.75889
	e9, e16,e29	0.71039
	e8, e18,e29	0.71623

## Data Availability

The data presented in this study are available on request from the author.
